# A Lightweight Insulator Defect Detection Model for Edge Computing Devices: PEBL-YOLO

**DOI:** 10.3390/s26134169

**Published:** 2026-07-02

**Authors:** Hao Wang, Jie Li, Qi Xing

**Affiliations:** School of Intelligent Science and Technology, Inner Mongolia University of Technology, Hohhot 010080, China; 13238484111@163.com (H.W.); xq123abc321@163.com (Q.X.)

**Keywords:** insulator defect detection, PEBL-YOLO, lightweight object detection, edge computing, YOLOv11

## Abstract

Insulators are critical insulation components in power transmission lines; however long-term exposure to adverse environmental conditions may threaten the safety and stability of power delivery. Existing studies primarily emphasize detection accuracy, while deployment efficiency and inference speed have received insufficient attention, limiting their applicability to CPU-based edge computing devices. To address these limitations, this paper proposes PEBL-YOLO, a lightweight model for insulator defect detection. The proposed model retains the external C3k2 structure of YOLOv11 while simplifying its internal bottleneck module, in which PConv is embedded to improve spatial feature extraction and fusion efficiency. In the neck, the original Path Aggregation Feature Pyramid Network (PAFPN) is reconstructed by integrating a Bidirectional Feature Pyramid Network (BiFPN) with Efficient Channel Attention (ECA), enabling more effective aggregation of multi-scale features and stronger focus on defect-related regions with minimal parameter increase. Moreover, a lightweight shared decoupled detection head is designed to decouple classification and regression branches. By combining parameter sharing with Group Normalization (GN) the detection head further reduces model complexity while maintaining accurate localization capability. Experimental results show that PEBL-YOLO contains only 1.68 M parameters. It achieves Precision, Recall, mAP@0.5, and mAP@0.5:0.95 of 95.0%, 92.1%, 94.4%, and 53.6%, respectively. These results demonstrate that PEBL-YOLO achieves a favorable trade-off between detection accuracy and parameter efficiency, providing a practical solution for lightweight insulator defect detection in edge computing scenarios.

## 1. Introduction

With the rapid expansion of modern infrastructure and urban construction, the operational reliability of power transmission systems has become increasingly important. During long-term operation, insulators are vulnerable to contamination-induced flashover, aging, and mechanical damage, which may eventually lead to power line faults [1]. Early insulator inspection methods mainly relied on handcrafted feature extraction. Although these methods achieved some success, their limited robustness and generalization capability have become increasingly apparent under complex inspection environments and diverse application scenarios.

In recent years, deep learning-based object detection has achieved remarkable progress in computer vision and has been widely applied to fields such as medical imaging and autonomous driving. Among these methods, the YOLO series, built upon convolutional neural networks (CNNs), has attracted considerable attention owing to its favorable balance between detection speed and accuracy [2]. Existing object detection algorithms can generally be categorized into two-stage and single-stage detectors. The first category includes two-stage detection algorithms, such as R-CNN [3], Fast R-CNN [4], Mask R-CNN [5], and Cascade R-CNN [6]. These methods first generate candidate regions and then perform classification and refined localization on the selected proposals. Subsequently, Liu et al. proposed a denoising feature pyramid network to enhance Transformer R-CNN, thereby achieving high-precision object detection [7]. Hang et al. extended the Faster R-CNN framework for few-shot object detection by introducing multi-level decoupled gradient layers, which improved detection accuracy under limited training samples [8]. Although two-stage detectors generally achieve high accuracy, they usually involve a large number of parameters and relatively slow inference speed, which limits their applicability in real-time and resource-constrained scenarios.

In contrast, single-stage detectors, such as SSD [9], RetinaNet [10], and the YOLO series [11], eliminate the candidate-region generation stage. Instead, they directly predict object categories and bounding-box locations through dense prediction, thereby reducing computational latency and making them more suitable for real-time detection. However, most YOLO-based studies have been optimized for natural images captured from ground-level perspectives, where object features are relatively distinct. With the continuous evolution of the YOLO series, numerous studies have further improved its architecture, focusing on lightweight implementation and enhanced object detection performance.

Meanwhile, attention mechanisms in deep learning have continued to evolve, further improving the representation capability and detection accuracy of object detection models. Originally developed for natural language processing, attention mechanisms have gradually been extended to computer vision, enabling models to focus more effectively on task-relevant visual regions. The introduction of Transformer-based architectures, such as Vision Transformer (ViT), into visual tasks has further demonstrated the effectiveness of attention mechanisms in complex scenarios [12]. For example, Zhu et al. introduced a Transformer prediction head into TPH-YOLOv5 to enhance global contextual modeling and improve object feature representation [13].

However, detection tasks in power equipment inspection exhibit task-specific characteristics and deployment constraints. Tao et al. proposed a two-stage detection method for insulator defect detection [14]. By employing data augmentation strategies such as Gaussian blurring and affine transformation, they improved model generalization and increased detection accuracy from 0.91 to 0.96. However, this method also substantially increased the number of parameters and training cost, thereby limiting its practical deployment. Subsequent studies further improved insulator detection methods by reducing overfitting, increasing inference speed, and enhancing small-object detection capability while maintaining comparable accuracy [15,16,17]. Wang et al. proposed an insulator detection model based on Darknet53 and Siamese networks to achieve fine-grained insulator extraction and improve detection accuracy [18].

Nevertheless, practical insulator inspection often faces challenges such as small target size, complex and variable backgrounds, high computational demand, and model complexity. Although existing improvements can enhance detection accuracy, they often increase model complexity and computational burden. For example, an attention-based backbone feature extraction network and a weighted feature fusion method were proposed to mitigate complex background interference and improve the identification of small insulators and defects [19]. However, with a model size of 227 MB and 59,622,929 parameters, this method is difficult to deploy on embedded devices and requires substantial memory resources.

Therefore, to better balance detection performance and practical deployment, particularly in unmanned aerial vehicle (UAV)-based inspection scenarios, researchers have explored lightweight detection models. Liu et al. introduced an improved YOLOv4-tiny for lightweight implementation [20]. By integrating a self-attention mechanism and a spatial pyramid pooling module, their method achieved higher detection accuracy than the original YOLOv4-tiny while maintaining comparable detection speed. Some studies have incorporated GhostNet modules to reduce the number of parameters and model size [21,22], while attention mechanisms have been introduced to improve the accuracy of lightweight models. In addition, Chen et al. proposed PConv based on a spatial mixing strategy, which reduces redundant computation while better preserving feature representation capability than Ghost-based operations [23].

In several studies [24,25], individual network modules were modified to reduce model size. Although these models achieved noticeable improvements, their FLOPs remained relatively high, indicating that computational efficiency was still insufficient. Weng et al. developed an improved lightweight network based on YOLOv5 for real-time insulator fault detection on mobile devices [26]. Li et al. designed a lightweight convolutional module, termed ECA-GhostNet-C2f (EGC), and constructed the EGC-CSPGhostNet backbone network for insulator defect detection [27]. However, lightweight convolutional operations may introduce accuracy degradation and may still be unsuitable for CPU-based edge computing devices under strict resource constraints. Zhang et al. introduced a lightweight attention mechanism to enable the model to focus more effectively on insulator features in both channel and spatial dimensions, thereby reducing the false negative rate [28]. However, their model still contains a relatively large number of parameters and exhibits high computational complexity, which limits its deployment on UAV inspection devices.

The novelty of this study does not lie in proposing an entirely new basic operator but in the structured reorganization and collaborative optimization of existing modules for the specific task of insulator defect detection, thereby achieving a better balance among detection accuracy, parameter count, and computational complexity. To address the challenges of small targets, complex backgrounds, and lightweight deployment in UAV-based inspection scenarios, this paper proposes PEBL-YOLO, an improved lightweight detection model built upon YOLOv11. The main contributions of this paper are summarized as follows:

1. A C3k2_PConv feature refinement structure is designed for the neck network. Different from directly replacing the backbone with a lightweight network, PConv is embedded into the internal bottleneck of C3k2 while maintaining the external residual structure, which reduces redundant spatial convolution and preserves stable feature transmission during multi-scale fusion
2. A BiFPN-ECA collaborative fusion mechanism is constructed. BiFPN is used to adaptively fuse features from different scales, while ECA recalibrates the fused channel responses, enabling the model to enhance defect-related features and suppress background interference with negligible parameter increase.
3. A lightweight shared detail-enhanced decoupled detection head is developed. By combining parameter sharing, GroupNorm, and detail-enhanced convolution, the proposed head reduces repeated branch parameters and improves the representation of edge and texture details, which is beneficial for small insulator defect localization.

## 2. Materials and Methods

**Figure 1:**
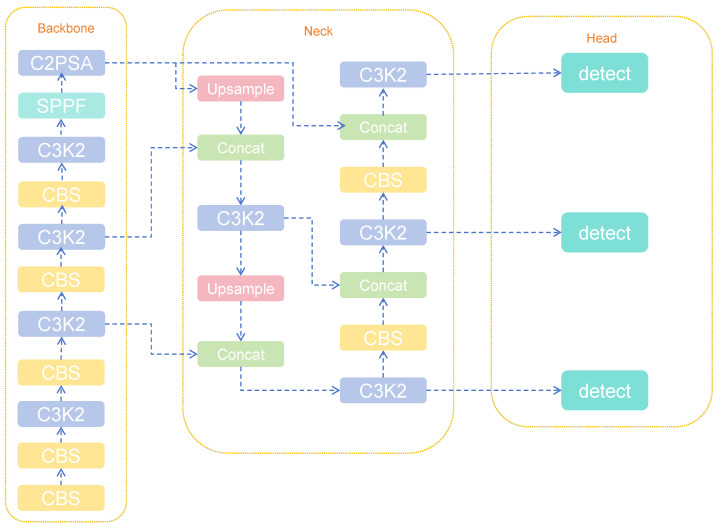
YOLOv11 architecture diagram.

In this study, we develop our method based on YOLOv11. YOLOv11 is a new-generation object detection framework developed by Ultralytics, introducing architectural and training-strategy improvements over previous YOLO versions [29]. Specifically, YOLOv11 replaces the previous C2f module with the C3k2 module and introduces the C2PSA module after pyramid pooling, thereby further improving feature extraction capability. The architecture of YOLOv11 is illustrated in Figure 1.

**Figure 2:**
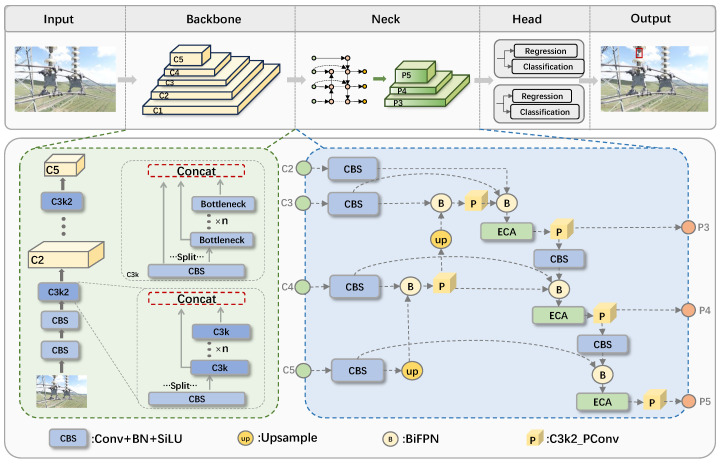
PEBL-YOLO architecture diagram.

To improve the model’s ability to detect insulator defects and facilitate its deployment, this paper proposes a lightweight PEBL-YOLO model. The paper retains the backbone network of YOLO11 whilst modifying the internal feature extraction, as shown in Figure 2.

First, the input image is processed by the backbone network to extract multi-level feature maps, denoted as C1, C2, C3, C4, and C5, which encode hierarchical spatial details and semantic representations. These feature maps are subsequently fed into the neck network to generate the multi-scale outputs P3, P4, and P5. To ensure that PEBL-YOLO maintains detection accuracy while achieving a lightweight design, the backbone is not aggressively lightweighted. Since the backbone is responsible for establishing the fundamental semantic representation of the input image, excessive lightweight modification at this stage may lead to the degradation or loss of critical visual information. Therefore, the proposed framework preserves the integrity of backbone feature extraction to maintain sufficient feature depth, diversity, and discriminative capacity.

In the neck network, the conventional concatenation operation used in YOLO is replaced with BiFPN [30] to enhance multi-scale feature fusion. Conventional concatenation simply stacks features along the channel dimension and treats inputs from different scales equally, whereas BiFPN introduces learnable fusion weights to model the relative contributions of features from different network levels. This mechanism enables the model to adaptively regulate information flow during training: for small objects, greater emphasis can be placed on shallow features containing detailed spatial cues, whereas for large objects, deeper features with stronger semantic abstraction can be assigned higher importance. Subsequently, the lightweight C3k2_PConv operator is introduced into the neck network during multi-scale feature fusion. By applying spatial convolution to only a subset of channels, C3k2_PConv effectively reduces computational redundancy in the feature fusion process.

After multi-scale fusion, an ECA module is further incorporated following BiFPN to refine the fused feature representations. Specifically, after BiFPN performs cross-scale alignment and feature aggregation, the fused multi-level features are passed to ECA for channel-wise recalibration [31]. Compared with the fully connected structure adopted in SENet [32], ECA captures local cross-channel interactions through one-dimensional convolution, without introducing fully connected layers. With negligible parameter overhead, the network can selectively emphasize informative channels and suppress less relevant responses. Furthermore, by highlighting salient channel-level responses and generating recalibrated feature maps, the model improves detection performance with minimal computational cost. Finally, a lightweight shared decoupled head is adopted to separate classification and localization tasks while sharing parameters across prediction branches. This design improves localization accuracy through task decoupling and substantially reduces model size through parameter sharing.

### 2.1. C3k2_PConv Lightweight Spatial Mixing and Feature Retention

**Figure 3:**
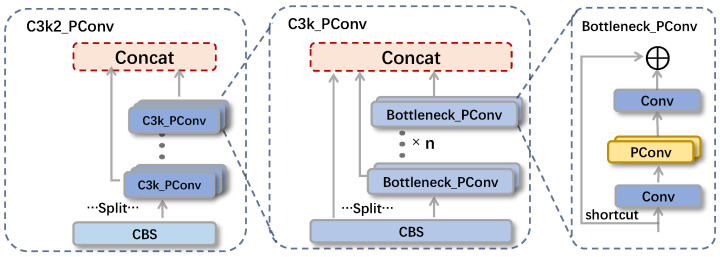
C3k2_PConv Module.

The core innovation of C3k2_PConv lies in the restructuring of its internal bottleneck architecture. This paper introduces partial convolution (PConv) to replace traditional full-channel convolution. As shown in Figure 3, the operational mechanism of PConv follows the logic outlined below:

The Neck stage involves a significant amount of cross-scale concatenation and feature alignment, which often results in severe channel redundancy. By using PConv at this stage, only a subset of the input feature maps undergoes convolution, thereby significantly reducing the computational cost of the model while maintaining detection accuracy.

1. At the operator level, PConv employs a channel-splitting strategy, performing 3×3 convolution only on $\frac{1}{n}$ of the active channels, while the remaining inactive channels are directly passed through. This design greatly suppresses the feature-overlap redundancy commonly observed in the Neck stage.
2. At the unit level, this paper constructs Bottleneck_PConv centered on PConv. Specifically, the input feature *X* is split into two parts along the channel dimension, namely $X_{\mathrm{conv}}\in R^{\frac{C}{n}\times H\times W}$ and $X_{\mathrm{untouched}}\in R^{(C-\frac{C}{n})\times H\times W}$, where *n* denotes the block factor. In this paper, PConv is embedded into the Bottleneck_PConv architecture to replace the conventional full-channel 3×3 convolution. This design performs partial convolution only on $X_{\mathrm{conv}}$ to extract the required contextual information, and its computational process is formulated as follows:

(1) $$Y_{\mathrm{conv}}={\mathrm{Conv}}_{1\times 1}\left({\mathrm{PConv}}_{3\times 3}\left({\mathrm{Conv}}_{1\times 1}\left(X_{\mathrm{conv}}\right)\right)\right),$$

(2) $$Y=\mathrm{Concat}\left(Y_{\mathrm{conv}},X_{\mathrm{untouched}}\right).$$

The first ${\mathrm{Conv}}_{1\times 1}$ layer is used for channel compression to reduce the scale of subsequent operations. The PConv stage serves as an intermediate layer for lightweight spatial feature extraction, while the second ${\mathrm{Conv}}_{1\times 1}$ layer restores the channel dimension. Finally, the original input and the processed features are fused through a residual connection. This design ensures that the model maintains stable gradient flow in deep layers while significantly reducing the number of parameters, thereby avoiding information loss caused by excessive lightweight processing.

3. At the functional abstraction level, this paper integrates multiple Bottleneck_PConv modules into the C3k2 framework. Unlike traditional concatenation, the features generated by BiFPN possess higher semantic density. C3k2_PConv efficiently refines these weighted-fused features through its lightweight architecture, thereby significantly optimizing inference latency in the Neck stage.

### 2.2. BiFPN + ECA Fusion Mechanism

In insulator inspection, although C3k2_PConv significantly reduces FLOPs through local convolution, this non-full-range feature processing mechanism may also dilute feature representation while improving efficiency. Furthermore, local convolution restricts the flow of spatial information across different channels, resulting in a large number of feature maps after multi-scale fusion but with limited feature quality. Traditional concatenation-based fusion indiscriminately mixes useful signals from P3 to P5 layers with irrelevant background noise, causing small-object features to be weakened under the strong semantic influence of larger objects.

**Figure 4:**
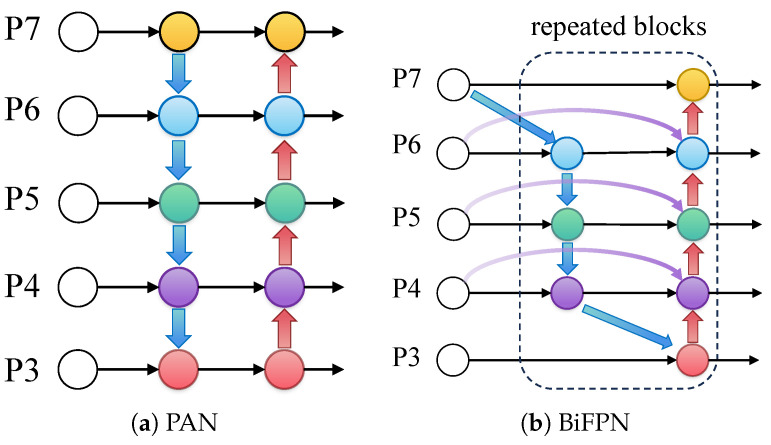
Comparison of PAN and BiFPN structures. Different colors distinguish feature levels, blue and red arrows indicate the top-down and bottom-up fusion paths, respectively, and the dashed box denotes repeated BiFPN blocks.

To address the limitations of lightweight operators in feature extraction and further enhance the fluidity of multi-scale features, this paper introduces an improved Bidirectional Weighted Feature Pyramid Network (BiFPN) following C3k2_PConv. Unlike PAN, as shown in Figure 4, PAN employs a uniform feature sampling strategy, which may introduce bias during cross-scale feature fusion and lead to the loss of small-scale defect features. The proposed BiFPN effectively alleviates this problem. Specifically, BiFPN simplifies PANet by removing nodes with only a single input, thereby reducing computational complexity while maintaining effective feature fusion.

Taking the fourth layer P4 as an example, where P3, P4, and P5 are used in this paper, the two fused features of BiFPN are formulated as follows:

(3) $$P_{4}^{\mathrm{td}}=\mathrm{Conv}\left(\frac{w_{1}\cdot P_{4}^{\mathrm{in}}+w_{2}\cdot \mathrm{Resize}\left(P_{5}^{\mathrm{in}}\right)}{w_{1}+w_{2}+\varepsilon }\right),$$

(4) $$P_{4}^{\mathrm{out}}=\mathrm{Conv}\left(\frac{w_{1}^{\prime }\cdot P_{4}^{\mathrm{in}}+w_{2}^{\prime }\cdot P_{4}^{\mathrm{td}}+w_{3}^{\prime }\cdot \mathrm{Resize}\left(P_{3}^{\mathrm{out}}\right)}{w_{1}^{\prime }+w_{2}^{\prime }+w_{3}^{\prime }+\varepsilon }\right).$$

In the equations, $P_{4}^{\mathrm{in}}$ denotes the input feature of the fourth layer, $P_{4}^{\mathrm{td}}$ denotes the intermediate feature of the fourth layer in the top-down path, and $P_{4}^{\mathrm{out}}$ denotes the output feature of the fourth layer in the bottom-up path. Moreover, $w_{i}$ and $w_{i}^{\prime }$ denote trainable fusion weights, and ε is a small constant used to ensure numerical stability.

Depending on the different objectives of upsampling and downsampling, BiFPN can effectively fuse, combine, and propagate insulator defect features across different feature scales. Since inputs with different resolutions contribute unequally to the fused output, BiFPN introduces learnable weights to adaptively adjust their importance. The fast normalized fusion strategy is expressed as follows:

(5) $$O=\sum_{i}\frac{w_{i}}{\varepsilon +\sum_{j}w_{j}}\cdot I_{i},$$

where $w_{i}$ denotes the weight learned by the network and $I_{i}$ represents the input feature map.

Through the bidirectional weighted fusion of BiFPN, the model achieves multi-scale feature aggregation at the macro level. However, the fused feature maps often exhibit channel-wise information redundancy, and the contribution, namely sensitivity, of different channels to the object detection task varies. Therefore, an efficient attention mechanism is required to further explore latent inter-channel correlations and suppress background noise.

**Figure 5:**
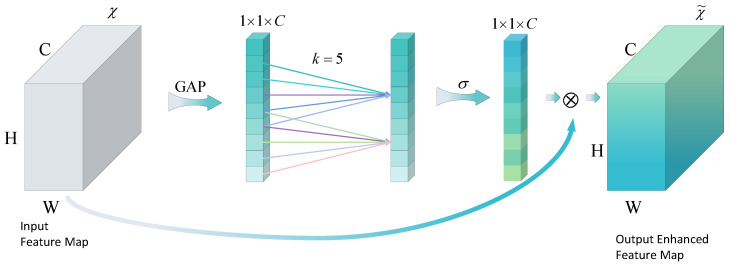
ECA Module.

Based on this motivation, the Efficient Channel Attention (ECA) module is introduced to refine the fused features. Unlike attention mechanisms that rely on dimensionality reduction, ECA avoids channel compression and employs one-dimensional convolution to promote local cross-channel interaction. Specifically, ECA first applies Global Average Pooling (GAP) to each channel feature to generate a global channel descriptor. Then, a one-dimensional convolution with kernel size *k* is applied to the global descriptor to capture local dependencies among adjacent channels. Through this process, the feature channels are adaptively recalibrated, allowing channels with stronger contributions to defect detection to receive higher weights while suppressing redundant background responses.The structure of the ECA module is illustrated in Figure 5.

By capturing cross-channel interaction information, ECA enhances object saliency during multi-scale feature fusion and improves the recognition capability for defects of different scales. Moreover, the ECA mechanism is simple and efficient, introducing almost no additional parameters. To achieve adaptive cross-channel interaction without manually introducing extra hyperparameters, ECA determines the convolution kernel size *k* according to the number of channels *C* as follows:

(6) $$k=\psi \left(C\right)={\left|\frac{\log_{2}\left(C\right)}{\gamma }+\frac{b}{\gamma }\right|}_{\mathrm{odd}},$$

where *C* denotes the number of channels, γ and *b* are predefined constants used to control the mapping relationship, and ${\left|\cdot \right|}_{\mathrm{odd}}$ indicates that the nearest odd number is selected. Overall, ECA has limited influence on inference speed while improving feature discrimination, thereby helping the model maintain both detection accuracy and computational efficiency.

### 2.3. LSDECD Lightweight Shared Decoupling Head

**Figure 6:**
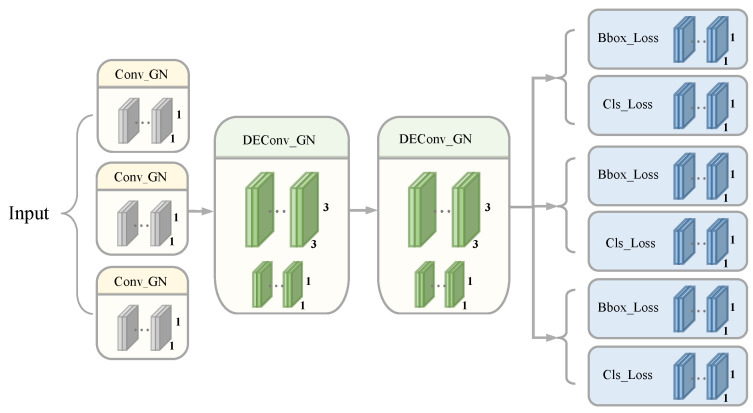
LSDECD Module.

To alleviate the computational burden of the original YOLOv11 detection head, a lightweight Shared-Decoupled Detection Head (LSDECD) is adopted in this work. By enabling feature sharing between classification and regression branches, LSDECD effectively reduces parameter redundancy and computational cost while maintaining detection performance. The overall architecture of the LSDECD detection head is shown in Figure 6.

The LSDECD detection head is developed based on LSCD, which improves object detection accuracy and efficiency while maintaining a lightweight structure. Specifically, LSDECD utilizes Group Normalization (GroupNorm) and shared convolution for efficient feature modeling. GroupNorm is independent of batch size and can maintain stable performance even under small-batch training conditions. By performing group normalization, it better preserves inter-channel relationships and enhances the robustness of the model.

**Figure 7:**
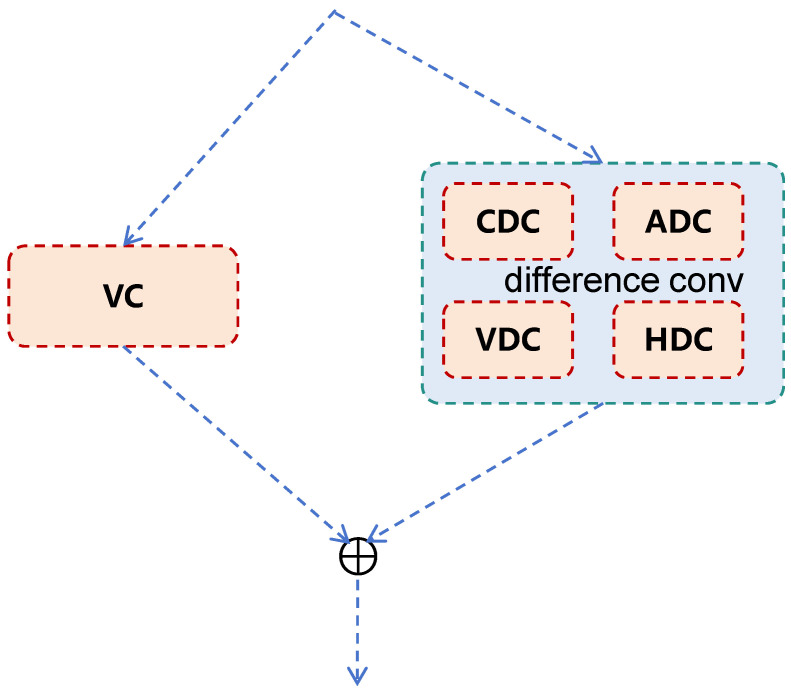
DEConv module.

Different from traditional independent convolution, shared convolution adopts a parameter reuse mechanism to maintain weight consistency across the global space, enabling more precise extraction of local features while preserving the lightweight property of the model. Furthermore, LSDECD introduces Detail Enhancement Convolution (DEConv) in the shared convolution stage. DEConv [33] consists of five convolutional branches that perform feature extraction in parallel. As shown in Figure 7, the structure contains five branches: vanilla convolution (VC), central difference convolution (CDC), angular difference convolution (ADC), horizontal difference convolution (HDC), and vertical difference convolution (VDC).

The CDC and ADC are responsible for capturing local defect features, while HDC and VDC enhance the edge and contour information of insulator defects. The DEConv layer takes the corresponding features extracted by the five convolutional kernels as input and produces the final output by summing their results, as formulated below:

(7) $$F_{\mathrm{out}}=\mathrm{DEConv}\left(F_{\mathrm{in}}\right)=\sum_{i=1}^{5}\left(F_{\mathrm{in}}*K_{i}\right)=F_{\mathrm{in}}*\left(\sum_{i=1}^{5}K_{i}\right)=F_{\mathrm{in}}*K_{\mathrm{cvt}}$$

(8) $$K_{\mathrm{cvt}}=\sum_{i=1}^{5}K_{i}$$

Here, $F_{\mathrm{out}}$ denotes the output feature, $F_{\mathrm{in}}$ denotes the input feature, $K_{i}$ represents the corresponding convolutional kernel in the five branches, ∗ denotes the convolution operation, and $K_{\mathrm{cvt}}$ denotes the equivalent 3×3 convolutional kernel obtained by stacking the five kernels. These modules are effective in extracting detailed information from minute defects and enhancing detection accuracy and robustness through multi-scale feature fusion and the integration of local and global information.

Finally, in the detection workflow, the feature maps are first fed into the 1×1 GN_Conv module through three-scale branches, where channel adjustment, feature normalization, and feature enhancement are performed. Subsequently, a two-layer DEConv_GN structure with shared weights is introduced to further improve the capability of capturing complex object features while reducing the number of parameters and computational overhead. Finally, the feature maps are passed into the decoupled prediction head. Each scale branch independently generates predictions for two tasks: the regression branch calculates the bounding box loss, denoted as Bbox Loss, to precisely localize the object, while the classification branch calculates the class loss, denoted as Cls Loss, to determine the object category.

## 3. Experiments

### 3.1. Experimental Environment

The experimental settings are summarized in Table 1. All experiments were conducted using the same training configuration to ensure a fair comparison.

### 3.2. Experimental Dataset

In real-world power grid environments, insulator inspection is confronted with a series of practical challenges, including densely distributed electrical components, frequent object occlusion, pronounced scale variation, and complex background interference arising from vegetation, transmission facilities, and surrounding power equipment. To better approximate practical inspection scenarios, this study constructs a customised insulator defect dataset by integrating the public IDID dataset with field inspection images collected from the Inner Mongolia ultra-high-voltage power grid. The dataset was divided into a training set, validation set, and test set at a ratio of 7:2:1, containing 1470, 403, and 227 images, respectively. The annotated categories include three classes: “broken”, “damage”, and “flashover”.

To further improve model generalization and enhance robustness against complex backgrounds, illumination variations, and imaging disturbances, several data augmentation strategies are introduced during the construction of the training samples, including image rotation, horizontal and vertical flipping, and brightness and contrast adjustment. The detailed augmentation parameters are reported in Table 2. After augmentation, the number of training images increased to 3045.

**Figure 8:**
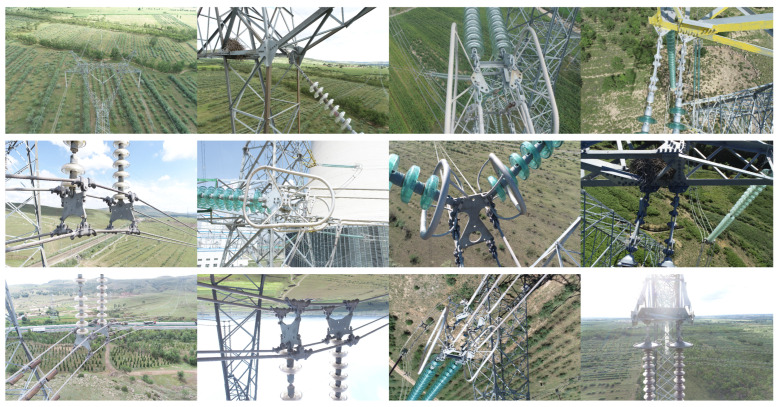
Customized dataset.

As illustrated in Figure 8, the constructed dataset combines standardized public data with real inspection images and covers multiple application scenarios, including transmission lines, substations, and typical outdoor inspection environments in Inner Mongolia. Compared with a single-source dataset, this integrated data construction strategy enhances scene diversity to a certain extent by incorporating insulator images captured under diverse backgrounds, shooting distances, object scales, and illumination conditions while also enriching the variety of insulator types and defect morphologies.

Specifically, the field images collected from the Inner Mongolia ultra-high-voltage power grid provide more engineering-oriented samples, including a certain number of small-scale, densely distributed objects and targets affected by strong background interference, which are relatively underrepresented in some public datasets. In contrast, the IDID dataset offers favorable openness and annotation foundations, thereby facilitating comparison with existing methods. Consequently, the fused dataset can more faithfully characterize conventional power grid inspection scenarios and, to some extent, narrows the gap between public benchmark data and practical engineering applications.

To further analyze the dataset characteristics, the class distribution and annotation information were statistically summarized, as shown in Table 3. The augmented dataset contains a total of 3695 images and 7391 defect instances, with 2825, 3875, and 691 annotated instances for the damage, flashover, and broken classes, respectively. The dataset exhibits certain class imbalance, reflecting the occurrence frequency of different defects in practical inspections.

**Figure 9:**
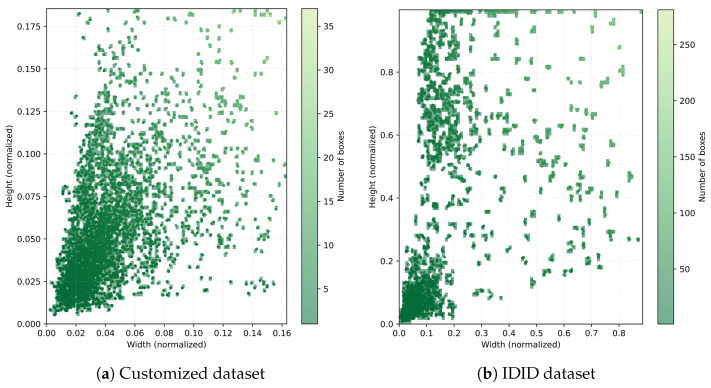
Representative images from the constructed insulator defect dataset.

Moreover, the dataset also shows variation in object sizes and scene distribution. Figure 9 intuitively illustrates the distribution of different target sizes in the self-collected dataset and the IDID dataset, showing that small and medium targets dominate in the self-collected dataset, while large targets are relatively few. To further characterize the target scale, the sizes of annotated objects were statistically analyzed. Table 4 shows that small, medium, and large targets account for 40.68%, 54.76%, and 4.56% of all annotated instances, respectively. The proportion of small and medium targets exceeds 95%, which is consistent with the actual imaging characteristics in UAV inspections and helps evaluate the model’s detection stability under multi-scale conditions.

### 3.3. Evaluation Metrics

To comprehensively evaluate the performance of the proposed model in insulator defect detection, Precision, Recall, mAP@0.5, and mAP@0.5:0.95 are employed as detection accuracy metrics. Meanwhile, the number of parameters (Parameters) and floating-point operations (GFLOPs) are introduced to quantify model size and computational complexity, thereby providing an indirect assessment of the model’s lightweight characteristics and its potential for resource-constrained deployment.

Precision and Recall are used to measure the correctness of model predictions and the completeness of target detection, respectively. They are defined as follows:

(9) $$\mathrm{Precision}=\frac{TP}{TP+FP}$$

(10) $$\mathrm{Recall}=\frac{TP}{TP+FN}$$

Here, TP denotes true positives, i.e., the number of positive samples correctly detected by the model; FP denotes false positives, i.e., the number of negative samples incorrectly predicted as positive; and FN denotes false negatives, i.e., the number of positive samples missed by the model. Accordingly, Precision and Recall measure the correctness and completeness of detection results, respectively.

Mean Average Precision (mAP) is adopted to comprehensively assess the model’s detection performance across all object categories. Specifically, mAP@0.5 denotes the mean Average Precision over all classes at an IoU threshold of 0.5, whereas mAP@0.5:0.95 represents the average mAP computed over IoU thresholds ranging from 0.5 to 0.95 at intervals of 0.05. The latter metric provides a stricter and more comprehensive evaluation of localization accuracy and detection robustness. The corresponding calculation is formulated as follows:

(11) $$AP_{i}=\int_{0}^{1}P_{i}\left(R\right)\;dR$$

(12) $$mAP=\frac{1}{C}\sum_{i=1}^{C}AP_{i}$$

where $P_{i}\left(R\right)$ denotes the functional relationship between Precision and Recall for the *i*-th category, $AP_{i}$ represents the area under the corresponding P–R curve, *C* is the total number of categories, and mAP is the average AP over all categories. Parameters characterize the number of learnable parameters in the model, whereas GFLOPs indicate the floating-point operations required for one forward inference. Together, these two metrics reflect the storage cost and computational burden of the model. The above evaluation metrics provide a quantitative basis for subsequent experimental analysis and model performance assessment.

### 3.4. Localization Comparative Experiments for Different Modules

#### 3.4.1. Localization Comparative Experiment of the C3k2_PConv Module

To further verify the effectiveness of the C3k2_PConv module at different feature levels, localization comparative experiments are designed around its replacement positions. The experimental results are shown in Table 5. Here, E1 denotes the baseline model without C3k2_PConv; E2, E3, and E4 represent the progressive introduction of this module into the P3, P3–P4, and P3–P5 feature levels, respectively; and E5 denotes the configuration in which all corresponding C3k2 structures in the Neck are replaced with C3k2_PConv.

As shown in Table 5, the overall detection accuracy improves progressively as the replacement scope of C3k2_PConv is expanded, while the number of parameters and GFLOPs remain relatively low. This result indicates that C3k2_PConv can effectively reduce redundant computation while enhancing feature representation, thereby achieving a favorable trade-off between detection accuracy and model lightweighting. Specifically, when C3k2_PConv is introduced only at the P3 level, the model already shows improved performance, suggesting that this module can enhance shallow-level details and improve the representation of small objects and edge-texture features. When the replacement scope is further extended to the P3–P4 and P3–P5 levels, model performance continues to improve, indicating that collaborative optimization across multi-scale feature layers strengthens the representation of targets with different sizes. Finally, when all corresponding C3k2 structures in the neck are replaced with C3k2_PConv, the model achieves the best performance while maintaining low parameter and computational complexity.

From a mechanistic perspective, this improvement does not arise from increased network complexity, but rather from more efficient feature extraction and computational resource allocation enabled by C3k2_PConv. Specifically, through the partial convolution mechanism, C3k2_PConv performs spatial convolution only on a subset of channels, while the remaining channels are propagated through lightweight paths. This design effectively reduces redundant computation and decreases the number of parameters. Meanwhile, this structure preserves critical feature information while reducing computational cost, thereby avoiding the degradation of representation capability commonly caused by excessive compression in conventional lightweight methods. Moreover, by selectively processing and fusing channel features, C3k2_PConv enhances discriminative information and suppresses redundant responses, thereby strengthening the coordination between shallow details and deep semantics during multi-scale feature fusion.

Therefore, the proposed C3k2_PConv module enables consistent improvements in detection accuracy without increasing model complexity and can even reduce it, thereby demonstrating a desirable trade-off between accuracy and efficiency. The experimental results further confirm that replacing all corresponding layers in the neck with C3k2_PConv fully exploits the advantages of this module. This strategy not only avoids additional computational burden but also further improves model performance, indicating that the full-scale replacement design is reasonable and effective, and that it provides a reliable basis for structural optimization in lightweight object detection models.

#### 3.4.2. Localization Comparative Experiment of the ECA Module

To verify the effect of the ECA attention mechanism at different feature levels, ECA modules are introduced after the fused outputs of P3, P4, and P5, respectively, and multiple ablation configurations are designed. The experimental results are reported in Table 6.

Overall, as the deployment scope of ECA is gradually expanded, Precision, Recall, and mAP all show a consistent upward trend. This demonstrates that ECA not only improves the reliability of detection results but also enhances the model’s object discovery capability and localization accuracy. Compared with the baseline model, introducing ECA into multi-scale feature layers markedly strengthens object discriminability and detection robustness.

More specifically, when ECA is introduced at only a single scale, model performance is improved, but the gain remains limited. This is mainly because a single-layer attention mechanism only reweights features at a specific scale. Although it can enhance feature responses and improve Precision to some extent, the lack of cross-scale information interaction limits its ability to perceive weak and small objects in complex backgrounds; consequently, the improvements in Recall and overall mAP remain limited. When ECA is jointly introduced at two scales, model performance further improves. This improvement mainly arises from more effective coordination among features at different levels: shallow features contain abundant spatial details and help improve the detection rate of small objects, thereby increasing Recall, whereas mid- and high-level features provide stronger semantic representations and help reduce false detections, thereby improving Precision. Hence, multi-scale ECA integration can simultaneously improve Precision and Recall to a certain extent, although incomplete feature coverage still leaves room for further performance enhancement.

Ultimately, when ECA is introduced after the fused features of all three scales, namely P3, P4, and P5, the model achieves the best results in terms of Precision, Recall, and mAP. This is because ECA can perform unified modeling of multi-scale fused features and adaptively adjust channel weights to emphasize informative responses across different scales while suppressing background noise and redundant activations. Specifically, enhanced discriminative responses help reduce false positives, strengthened weak small-object features help mitigate missed detections, and improved consistency of multi-scale feature representations further enhances localization stability. As a result, the model achieves improvements in detection accuracy, object completeness, and overall localization precision.

It is worth noting that, across all experimental settings, GFLOPs remain unchanged, while the number of parameters increases only marginally. This indicates that ECA, as a lightweight attention mechanism, introduces negligible additional computational overhead while delivering notable performance gains. Therefore, uniformly introducing ECA after P3–P5 multi-scale fusion represents an effective design strategy that improves Precision, Recall, and mAP without increasing model complexity. This result also suggests that introducing lightweight attention mechanisms after multi-scale feature fusion is an effective structural optimization strategy and provides useful guidance for the design of future lightweight object detection models.

### 3.5. Ablation Experiments

The localization of C3k2_PConv and ECA has been analyzed separately, and their optimal configurations across multi-scale feature layers have been determined. On this basis, a comprehensive ablation experiment is conducted to further verify the synergistic effects among the improved modules. The results are shown in Table 7.

Table 7 reports the ablation results of different modules. Overall, compared with the Baseline, the complete model improves Precision, Recall, mAP@0.5, and mAP@0.5:0.95 by 3.12%, 4.18%, 2.72%, and 11.54%, respectively, while reducing GFLOPs by 7.94% and parameters by 34.75%. The improvement in Precision indicates a reduction in false detections, the improvement in Recall suggests more complete target discovery, and the substantial gain in mAP@0.5:0.95 demonstrates enhanced detection stability and robustness under stricter localization criteria. These results show that the improvement in model performance is not driven by increased computational complexity, but by enhanced feature representation quality and more efficient information utilization.

From the single-module results, different modules address distinct performance bottlenecks. C3k2_PConv increases mAP@0.5:0.95 by 4.90%, which is more pronounced than the 1.28% gain obtained by introducing only BiFPN. This indicates that C3k2_PConv improves localization accuracy by reducing redundant computation and strengthening key feature representation. Although BiFPN yields an approximately 1.24% improvement in Recall, its enhancement of Precision and high-IoU performance remains limited, implying that multi-scale fusion alone is insufficient to effectively suppress background interference. ECA further increases mAP@0.5:0.95 by 6.82% on the basis of BiFPN, revealing its clear advantage in enhancing discriminative features. Meanwhile, LSDECD improves Recall and mAP@0.5:0.95 by 3.32% and 6.68%, respectively, and the magnitude of improvement is notably larger than that achieved by BiFPN alone, indicating that detection-head optimization plays a more direct role in improving target completeness.

Further analysis of the combined configurations reveals clear synergy and interdependence among the modules. First, after combining BiFPN with C3k2_PConv, mAP@0.5:0.95 increases by an additional 5.25% compared with introducing BiFPN alone, while GFLOPs decrease, suggesting that C3k2_PConv can reduce redundant computation and improve feature representation quality on the basis of multi-scale fusion. Moreover, compared with Baseline+A+B, adding ECA further improves Precision, indicating that post-fusion feature selection helps suppress background interference and reinforce effective features. However, the combined design still relies on high-quality feature inputs. For example, when C3k2_PConv is absent, several metrics fluctuate, suggesting that if redundant information remains in the features, the effects of ECA and LSDECD cannot be fully exploited. This further confirms that feature quality is a prerequisite for subsequent modules to perform effectively. From the perspective of combined experiments, these modules are not merely additive; rather, they exhibit explicit complementarity. If any component is removed, Precision, Recall, or mAP will degrade to varying degrees, demonstrating the indispensable contribution of each module.

The ablation results demonstrate that the proposed modules are not simply stacked as independent plug-in components, but rather form a complementary and progressive optimization framework. Through a sequence of coordinated optimizations, the proposed model achieves more stable detection performance in insulator inspection scenarios characterized by complex backgrounds, dense small targets, and pronounced scale variations. It also realizes a favorable balance between detection accuracy and lightweight design, thereby demonstrating promising engineering deployment value.

### 3.6. Comparative Experiments

The proposed model is a lightweight object detection framework specifically designed for insulator defect detection. To comprehensively evaluate the effectiveness and engineering applicability of the proposed method, comparisons are conducted with the baseline model, representative general-purpose object detectors, and recent task-specific lightweight detection models. The general-purpose detectors include YOLOv5n, YOLOv8n, YOLOv10, YOLOv11n, YOLOv12, YOLOv26, and Transformer-based DETR and RT-DETRv2, while MIF-YOLO, MobileNetV3-YOLO and EfficientDet-D0 are selected as a task-specific lightweight comparison model. All models are trained and tested on the same dataset under identical experimental settings to ensure a fair comparison.

The quantitative results in Table 8 demonstrate substantial differences among detection models in terms of detection accuracy and computational complexity, whereas the proposed method achieves the best overall performance. Specifically, the proposed model attains Precision, Recall, mAP@0.5, and mAP@0.5:0.95 values of 0.964, 0.940, 0.960, and 0.564, respectively, outperforming all comparison models. Compared with mainstream lightweight detectors, namely YOLOv8n, YOLOv10, and YOLOv11n, the proposed method improves mAP@0.5 by 1.5%, 2.1%, and 2.3%, respectively, and improves mAP@0.5:0.95 by 3.9%, 4.5%, and 5.8%, respectively. These results indicate that the proposed model can extract more sufficient discriminative features in scenarios involving complex backgrounds, multi-scale variations, and small defect targets, thereby improving both target recognition and localization performance. Compared with the task-specific lightweight model MIF-YOLO, the proposed method achieves gains of 0.6% in mAP@0.5 and 0.8% in mAP@0.5:0.95. Moreover, it outperforms MobileNetV3-YOLO and EfficientDet-D0 by 2.4% and 2.0% in mAP@0.5, and by 5.3% and 4.1% in mAP@0.5:0.95. These results demonstrate that the proposed architectural design provides consistent performance improvements even when compared with strong lightweight competing models.

The comparison across different architectural paradigms further reveals the close relationship between model structure and task characteristics. Although DETR and RT-DETRv2 are capable of modeling global dependencies, they do not show clear accuracy advantages in the insulator defect detection task. DETR achieves only 0.894 mAP@0.5, while requiring 115.2 GFLOPs and 41.84M parameters. RT-DETRv2 improves the mAP@0.5 to 0.935; however, its computational cost and parameter count remain as high as 60.0 GFLOPs and 20.11M, which are substantially larger than those of lightweight YOLO-based models. In contrast, the YOLO series benefits from convolutional structures that are more suitable for local texture representation and multi-scale feature fusion, thereby showing better adaptability to inspection images with dense small targets and complex backgrounds. YOLOv26 achieves 0.946 mAP@0.5 with only 5.2 GFLOPs, indicating favorable computational efficiency, but its detection accuracy is still lower than that of the proposed method. MobileNetV3-YOLO reduces the model size by adopting a lightweight backbone, but its mAP@0.5:0.95 is only 0.511, suggesting that simply relying on a lightweight backbone may weaken the feature representation capability for complex defective regions. EfficientDet-D0 achieves 0.940 mAP@0.5 through multi-scale feature fusion, yet its mAP@0.5:0.95 remains inferior to that of the proposed method. These results indicate that the general evolution of model architectures alone is insufficient to simultaneously satisfy the requirements of high accuracy and lightweight deployment in insulator defect detection.

The analysis of model complexity indicates that the proposed method reinforces its lightweight advantage while achieving the highest detection accuracy. The model contains only 1.69M parameters, which is substantially lower than those of YOLOv8n (3.14 M), YOLOv11n (2.58 M), MIF-YOLO (2.33 M), MobileNetV3-YOLO (2.10 M), and EfficientDet-D0 (3.90M), corresponding to reductions of approximately 46.2%, 34.5%, 27.5%, 19.5%, and 56.7%, respectively. In terms of computational cost, the proposed method requires 5.8 GFLOPs, lower than YOLOv8n, YOLOv10, and YOLOv11n, and comparable to YOLOv12n. Although EfficientDet-D0 has a lower GFLOPs value, its detection accuracy and practical inference speed remain inferior to the proposed model. Overall, the proposed method achieves the optimal detection performance under the constraints of minimal parameter count and relatively low computational cost, demonstrating an excellent balance among accuracy, model size, and computational efficiency.

In addition, to further evaluate the practical deployment efficiency of different models, the inference frame rate (FPS) was measured under the same testing environment. As shown in Table 8, although Transformer-based detectors have the advantage of global dependency modeling, their complex architectures lead to limited inference efficiency. Specifically, DETR and RT-DETRv2 achieve only 22.15 FPS and 48.32 FPS, respectively, making them difficult to satisfy the real-time requirements of inspection tasks. EfficientDet-D0 has a relatively low GFLOPs value; however, due to operations such as multi-scale feature fusion and feature resampling, its actual inference speed reaches only 128.54 FPS. In contrast, lightweight YOLO-based models generally exhibit higher inference efficiency, with MIF-YOLO and MobileNetV3-YOLO achieving 225.46 FPS and 231.38 FPS. The proposed method achieves the highest detection accuracy while reaching an inference speed of 284.16 FPS with only 1.69M parameters. These results further demonstrate its potential for real-time deployment in resource-constrained scenarios such as UAV inspection and edge computing.

To evaluate the stability of the proposed model, five repeated experiments were conducted under the same experimental settings. As shown in Table 9, the proposed method achieves an average mAP@0.5 of 0.960 with a standard deviation of only 0.001, and an average mAP@0.5:0.95 of 0.563 with a standard deviation of 0.003. These results indicate that the proposed model maintains stable detection performance across different runs.

#### Visualization of Detection Results

**Figure 10:**
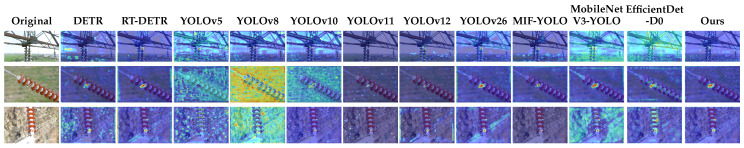
Heatmap comparison among different models.

This study employs Grad-CAM (Gradient-weighted Class Activation Mapping) visualization to compare the feature response regions of different models. This analysis further reveals, from the perspective of model interpretability, the attention mechanisms adopted by different detection methods during insulator defect recognition. Unlike quantitative metrics in tables, heatmaps can intuitively display the image regions emphasized by the model during prediction and help determine whether the model is affected by complex background factors such as towers, ground textures, and surrounding infrastructure. To ensure fairness of comparison, the same test samples are selected for visualization. The models involved in the comparison are consistent with those in the preceding comparative experiments, including multiple general-purpose object detection models, Transformer-based detectors, task-specific lightweight models, and the proposed method. The selected samples cover the three insulator defect categories in the dataset, enabling a comprehensive evaluation of the feature attention capability and background-interference resistance of different models under practical application conditions. Figure 10 presents the Grad-CAM heatmap comparisons for representative samples from the three defect categories.

Different detection models show distinct strengths in defect localization and background suppression. Transformer-based detectors, such as DETR and RT-DETR, tend to activate non-target regions under complex backgrounds, including towers, conductors, and surrounding textures. General-purpose YOLO models can capture defect-related regions, but their attention may become dispersed or spatially shifted when detecting small defects. Although lightweight models improve computational efficiency, their heatmap responses are often less continuous and stable. In contrast, the proposed method produces more discriminative feature responses, with activations consistently concentrated on insulator defects and adjacent informative regions. This demonstrates that the introduced feature enhancement and multi-scale fusion mechanisms can strengthen defect representation, suppress irrelevant background interference, and reduce false detections and localization errors in complex inspection scenarios.

Therefore, the proposed model can strengthen defect-related information during multi-scale feature interaction while suppressing interference from irrelevant background features, thereby establishing more reliable discriminative evidence in insulator images. The visualization results further demonstrate the comprehensive advantages of the proposed method in background-interference suppression and small-defect representation.

## 4. Conclusions

To address the challenges of small targets, complex backgrounds, and limited computational resources in UAV-based inspection scenarios, this study proposes a lightweight insulator defect detection method based on YOLOv11. By coupling BiFPN with ECA, the proposed model improves multi-scale feature aggregation while enhancing the discriminative representation of defect-related regions. Furthermore, PConv is embedded into the C3k2 structure to reduce redundant spatial convolution and strengthen lightweight feature extraction. A lightweight detection head, LSDECD, is also introduced to further reduce computational burden while preserving localization and classification performance. As a result, the proposed framework achieves an effective trade-off between detection accuracy and computational efficiency. Experimental results show that the proposed method outperforms multiple mainstream detection models in terms of mAP and other metrics while requiring lower computational complexity and fewer parameters, thereby validating its effectiveness and efficiency. The ablation experiments further confirm the synergistic contribution of each module to improving detection robustness in complex scenarios.

From an engineering application perspective, the proposed method provides a practically deployable solution for intelligent transmission-line inspection. Compared with traditional manual inspection and hand-crafted feature-based approaches, the proposed deep learning framework can locate insulator defects more efficiently and demonstrates stronger adaptability to complex inspection scenarios. Moreover, the model is designed with lightweight deployment in mind. Owing to its low parameter count, reduced computational complexity, and high inference speed, it shows promising potential for UAV platforms and resource-constrained edge-computing scenarios. However, real edge-device deployment still requires further validation.

Despite the favorable experimental results, several limitations remain. First, the current experiments are mainly conducted on existing datasets, and the generalization capability of the model under multi-source, cross-regional, and complex meteorological conditions requires further validation. Second, this study primarily focuses on static-image detection and has not fully exploited the temporal continuity and motion information contained in UAV video sequences. Third, although the proposed model exhibits favorable lightweight characteristics, its inference efficiency, resource consumption, and operational stability on real edge-computing platforms still need to be further evaluated. Future work will focus on practical engineering applications, including deployment and validation on representative edge-computing platforms and in real inspection scenarios, to further investigate the applicability and engineering value of the model in complex environments. In addition, more diverse and large-scale insulator inspection datasets will be constructed, and temporal modeling, domain adaptation, data augmentation, pruning, quantization, and knowledge distillation will be explored to further improve model robustness, inference efficiency, and practical deployability.Overall, the proposed lightweight detection framework achieves a favorable balance among detection accuracy, computational efficiency, and deployment feasibility, providing a promising technical solution for intelligent UAV-based inspection of transmission-line insulators.

## Figures and Tables

**Table 1:** Training and Environment Parameters.

Parameter	Setting
GPU	NVIDIA Tesla V100S-PCIE-32GB
Python/PyTorch/Ultralytics	3.9.19/1.11.0/8.3.9
Image Size (imgsz)	896 × 896
Epochs	150
Training Batch Size	8
Inference Batch Size	8
Optimizer	SGD
Learning Rate	0.01
Momentum	0.937
Weight Decay	0.0005
Random Seed	42
Conf	0.25
IoU/NMS Threshold	0.6
Inference Mode	FP32

**Table 2:** Parameter settings of data augmentation methods.

Augmentation Method	Parameter Setting
Image rotation	$-10^{\circ }$ to $+10^{\circ }$
Horizontal flipping	Probability = 0.5
Vertical flipping	Probability = 0.5
Contrast adjustment	0.7 to 1.3
Brightness adjustment	−40 to +40
Gaussian blur	3×3 or 5×5, probability = 0.5
Gaussian noise	Mean = 0, standard deviation ≈10, probability = 0.5
Color shift	−20 to +20

**Table 3:** Class Distribution Statistics of Dataset.

Class	Number of Images	Number of Instances
Damage	1856	2825
Flashover	1357	3875
Broken	613	691
Total	3695	7391

**Table 4:** Proportion of Targets by Size.

Metric	Value
Small objects	40.68%
Medium objects	54.76%
Large objects	4.56%

**Table 5:** Localization experiment results of the C3k2_PConv module.

Experiment	Position					P	R	mAP@0.5	mAP@0.5:0.95	GFLOPs	Parameters (M)
	16	19	23	27	31						
E1	×	×	×	×	×	0.958	0.917	0.946	0.543	6.2	1.75
E2	×	×	✓	×	×	0.960	0.920	0.956	0.543	6.0	1.74
E3	×	×	✓	✓	×	0.960	0.930	0.950	0.545	6.0	1.73
E4	×	×	✓	✓	✓	0.963	0.916	0.947	0.541	6.0	1.71
**E5**	✓	✓	✓	✓	✓	**0.964**	**0.940**	**0.960**	**0.564**	**5.8**	**1.69**

Note: The results of the final proposed configuration (E5) are highlighted in bold. A checkmark (✓) indicates that the corresponding position is selected for module insertion, whereas a cross (×) indicates that it is not selected.

**Table 6:** Localization comparative experimental results of ECA at different positions.

Experiment	Location			P	R	mAP@0.5	mAP@0.5:0.95	GFLOPs	Parameters
	22	26	30						
A1	×	×	×	0.935	0.934	0.955	0.543	5.8	1,685,162
A2	✓	×	×	0.954	0.907	0.947	0.536	5.8	1,685,293
A3	×	✓	×	0.956	0.903	0.941	0.536	5.8	1,685,293
A4	×	×	✓	0.959	0.927	0.949	0.540	5.8	1,685,293
A5	✓	✓	×	0.961	0.913	0.943	0.531	5.8	1,685,424
A6	✓	×	✓	0.962	0.932	0.954	0.551	5.8	1,685,424
A7	×	✓	✓	0.963	0.929	0.953	0.549	5.8	1,685,424
**A8**	✓	✓	✓	**0.964**	**0.940**	**0.959**	**0.564**	**5.8**	**1,685,555**

Note: A checkmark (✓) indicates that the ECA module is inserted at the corresponding location, whereas a cross (×) indicates that it is not inserted. Boldface highlights the final proposed configuration (A8) and its corresponding results.

**Table 7:** Ablation experiment results. A denotes the BiFPN multi-scale feature fusion module, B denotes the C3k2_PConv lightweight feature extraction module, C denotes the ECA channel attention mechanism, and D denotes the LSDECD detection head.

Model	P	R	mAP@0.5	mAP@0.5:0.95	GFLOPs	Parameters (M)
Baseline	0.935	0.902	0.934	0.506	6.3	2.58
Baseline + A	0.952	0.913	0.942	0.512	6.3	1.95
Baseline + B	0.955	0.922	0.944	0.530	6.1	2.40
Baseline + D	0.952	0.932	0.941	0.539	6.0	2.26
Baseline + A + B	0.955	0.928	0.959	0.539	5.8	1.86
Baseline + A + C	0.962	0.924	0.949	0.547	6.3	1.92
Baseline + A + D	0.957	0.933	0.949	0.543	6.2	1.85
Baseline + A + B + C	0.958	0.936	0.956	0.540	5.8	1.86
Baseline + A + B + D	0.955	0.934	0.955	0.543	5.8	1.69
Baseline + A + C + D	0.958	0.927	0.950	0.545	6.2	1.75
**Ours**	**0.964**	**0.940**	**0.959**	**0.564**	**5.8**	**1.69**

Note: Boldface highlights the final proposed model (Ours) and its corresponding results.

**Table 8:** Comparative experimental results of different detection models.

Model	P	R	mAP@0.5	mAP@0.5:0.95	GFLOPs	Parameters (M)	FPS
YOLOv5n	0.912	0.873	0.909	0.483	5.8	2.18	150.67
YOLOv8n	0.948	0.915	0.945	0.525	8.5	3.14	138.25
YOLOv10	0.946	0.893	0.939	0.519	6.5	2.27	183.76
YOLOv11n	0.935	0.902	0.937	0.506	6.3	2.58	214.89
YOLOv12n	0.930	0.873	0.928	0.499	5.8	2.51	164.42
YOLOv26	0.954	0.922	0.946	0.540	5.2	2.36	228.73
DETR	0.910	0.822	0.894	0.481	115.2	41.84	22.15
RT-DETRv2	0.938	0.900	0.935	0.515	60.0	20.11	48.32
MIF-YOLO	0.952	0.928	0.954	0.556	6.0	2.33	225.46
MobileNetV3-YOLO	0.939	0.896	0.936	0.511	4.7	2.10	231.38
EfficientDet-D0	0.944	0.905	0.940	0.523	2.5	3.90	128.54
**Ours**	**0.964**	**0.940**	**0.960**	**0.564**	**5.8**	**1.69**	**284.16**

Note: Boldface highlights the results of the proposed model (Ours).

**Table 9:** Results of repeated experiments.

Run	P	R	mAP@0.5	mAP@0.5:0.95
1	0.964	0.940	0.960	0.564
2	0.961	0.936	0.958	0.560
3	0.966	0.942	0.962	0.567
4	0.961	0.939	0.960	0.562
5	0.964	0.941	0.960	0.564
Mean ± Std	0.963 ± 0.002	0.940 ± 0.002	0.960 ± 0.001	0.563 ± 0.003

## Data Availability

The datasets generated and/or analyzed during the current study are available from the corresponding authors upon reasonable request.
